# Health service needs in the aftermath of natural disasters: Perspectives from 51 people living with Alzheimer's Disease and their family caregivers in Puerto Rico

**DOI:** 10.1002/alz70860_104088

**Published:** 2025-12-23

**Authors:** Alexandra Mendoza‐Graf, Gabriela Castro, Gilberto Ramos Valencia, Mariela Torres‐Cintrón, Ivonne Z. Jimenez Velazquez, Ilka Rios, Johan Hernandez Santiago, Beverly Weidmer, Marielena Lara, Justin W. Timbie

**Affiliations:** ^1^ RAND Corporation, Santa Monica, CA, USA; ^2^ University of Puerto Rico, School of Medicine, San Juan, PR, USA; ^3^ University of Puerto Rico, School of Medicine, San Juan, Puerto Rico, PR, USA; ^4^ RAND Corporation, Alexandria, VA, USA

## Abstract

**Background:**

People living with Alzheimer's disease (AD) have a greater risk of adverse health outcomes following natural disasters and public health emergencies because of disruptions in support from caregivers and greater difficulty accessing medical care. Hurricane Maria, the fifth largest hurricane in US history, struck Puerto Rico in September 2017 and caused more than 3,000 deaths. We assessed the health service needs of AD patients and their family caregivers that emerged during and after these disasters and identified opportunities to address unmet needs through improved policy.

**Method:**

We conducted 51 in‐person interviews between 12/2023 and 11/2024 with AD patients and their family caregivers living in Puerto Rico. Interviews were conducted in Spanish and were transcribed and translated to English. Interrater reliability was established using Cohen's kappa, and transcripts were analyzed using a directed content approach.

**Result:**

The most widely reported service needs included: (1) support with personal care services, which are disproportionately shouldered by family caregivers in Puerto Rico due to the lack of coverage of home health services by Medicare and Medicaid, (2) care for trauma‐related symptoms among AD patients, including loss of appetite, sleep disruption, and depression, (3) respite care (particularly adult day services) to provide relief to caregivers during the recovery period while also addressing patients’ social isolation that is exacerbated by disasters, (4) support for health‐related social needs including nutritional assistance during extended periods without electricity, (5) support with care coordination due to the added burden of caregivers having to pick up paper‐based prescriptions from doctor's offices and be responsible for sharing lab results with different providers, and (6) additional modes for communicating with providers following these disasters.

**Conclusion:**

AD patients and caregivers reported numerous service needs associated with recent disasters. Policies that build on these findings that could facilitate access to care include: expanding computerization of administrative processes to include specialist referrals and laboratory test result delivery, maintenance of telehealth within Medicare following the March 2025 sunset of pandemic flexibilities, enhanced Medicare and Medicaid financing to cover long‐term services and supports, and investments in Puerto Rico's electrical grid to improve reliability and support these delivery system improvements.

